# High FN1 expression is associated with poor survival in esophageal squamous cell carcinoma

**DOI:** 10.1097/MD.0000000000033388

**Published:** 2022-04-07

**Authors:** Junliang Ma, Shaolin Chen, Min Su, Wenxiang Wang

**Affiliations:** a Department of Thoracic Surgery, Affiliated Hospital of Zunyi Medical University, Zunyi, Guizhou, P.R. China; b Nursing Department, Affiliated Hospital of Zunyi Medical University, Zunyi, Guizhou, P.R. China; c Department of the 2nd Department of Thoracic Surgery, Hunan Cancer Hospital and The Affiliated Cancer Hospital of Xiangya School of Medicine, Central South University, Changsha, Hunan, P.R. China.

**Keywords:** esophageal squamous cell carcinoma, fibronectin 1, ROC curve

## Abstract

Esophageal cancer (EC) is a serious threat to human health. The expression of fibronectin 1 (FN1) in esophageal squamous cell carcinoma (ESCC) remains controversial. The purpose of this study was to elucidate the expression of FN1 in ESCC and to assess the value of FN1 in the prognosis of ESCC patients. 100 ESCC patients from January 2015 to March 2016 were recruited in this study. qRT-PCR and immunohistochemistry (IHC) were used to detect FN1 mRNA and protein expression. The correlation between FN1 expression levels and prognosis of ESCC patients was analyzed. The qRT-PCR results showed that the expression of FN1 mRNA was significantly higher in ESCC tumor tissues than in adjacent esophageal tissues (*P* < .01). IHC results showed that FN1 protein was expressed in both tumor cells and stroma. High expression of FN1 mRNA and FN1 protein in ESCC tumor tissues was significantly correlated with the depth of tumor invasion, lymph node metastasis and clinical stage of the tumor (*P* < .05). Survival analysis revealed that patients with higher FN1 mRNA and protein expression had significantly lower survival rates than those with lower FN1 mRNA or protein expression (*P* < .01). Multivariate cox regression analysis showed that high FN1 protein expression in ESCC tumor tissues was an independent risk factor for low survival in ESCC patients (*P* < .05). High expression of FN1 protein in ESCC tumor tissue is an independent poor prognostic factor. FN1 protein could be a potential target for the treatment of ESCC.

## 1. Introduction

Esophageal cancer (EC) is a lethal malignancy, ranking sixth in the global causes of cancer-related deaths and ninth in the incidence of all cancers.^[1]^ Esophageal squamous cell carcinoma (ESCC) is one of the major pathological subtypes in China,^[2,3]^ and its patients with advanced stages have a poor prognosis.^[4,5]^ Although great efforts have been made to improve diagnosis and treatment, there are still many difficulties in the early diagnosis, recurrence control and prognostic evaluation of ESCC.^[6,7]^ ESCC is related to gene regulatory network, and its pathogenesis is still not fully understood.^[5]^ Despite improvements in surgery and chemotherapy/radiotherapy, the prognosis of ESCC patients is still poor. Therefore, it is crucial for ESCC patients to find new biomarkers for early diagnosis and new treatment methods to improve the survival time of patients.

An important step in the formation of metastasis and disease progression is the invasion of tumor cells into the extracellular matrix.^[8]^ Fibronectin 1 (FN1) is a macromolecular structural glycoprotein that mediates various cell-extracellular matrix interactions and plays a vital role in cell adhesion, migration, growth and differentiation.^[9,10]^ Plasma and cytosolic fibronectin are the 2 main biological types of FN1.^[11]^ Plasma FN1 is synthesized by hepatocytes and released into the circulation, while cellular FN1 is involved in the formation of the extracellular matrix, which can be found in most tissues.^[12]^ However, the location of FN1 protein expression in tumor tissues and the relationship between FN1 protein expression levels and prognosis of ESCC patients remain controversial. Therefore, the aim of this study was to elucidate the expression level of FN1 mRNA and protein in ESCC and to assess the value of FN1 in the prognosis of ESCC patients. We present the following article in accordance with the TRIPOD reporting checklist.

## 2. Materials and methods

### 2.1. Tissue specimens

This study included 100 patients who underwent esophagogastrostomy from January 2015 to March 2016 at the 2nd Department of Thoracic of Hunan Cancer Hospital. All patients were diagnosed as ESCC and none of them underwent radiotherapy or chemotherapy before surgery. The tumor tissues and adjacent normal esophageal tissues were collected during the operation. The diagnosis of clinical staging based on UICC 2009 TNM staging system. Information of survival period was obtained through telephone or e-mail contact. This study was approved by the Institutional Ethics Committee of Hunan Cancer Hospital and was performed in accordance with the relevant regulations.

### 2.2. RNA extraction and quantitative real-time PCR (qRT-PCR)

Total RNA was extracted from tissues by Trizol reagent (Ambion) and reverse transcribed by GoScript reverse transcription system (GeneCopoeia). qRT-PCR was implemented using All-in-One qPCR Mix (GeneCopoeia). GAPDH was used as internal control gene. FN1-specifc oligonucleotide primers were designed as follows: FN1 forward, 5´-GAGAATAAGCTGTACCATCGCAA-3´, reverse, 5´-CGACCACATAGGAAGTCCCAG-3´; GAPDH forward, 5´-ACAGCCTCAAGATCATCAGC -3´, reverse, 5´-GGTCATGAGTCCTTCCACGAT-3´. PCR amplification to quantify the levels of FN1 mRNA and GAPDH mRNA was performed using a Light cycler 96 Real-Time PCR system.

The amplification conditions consisted of initial denaturation at 95°C for 10 minutes followed by 40 cycles of denaturation at 95°C for 10 seconds, annealing at 60°C for 20 seconds, and elongation at 72°C for 15 seconds. Expression of FN1 mRNA was calculated by dividing the quantity of FN1 mRNA with the quantity of GAPDH mRNA (2^−ΔCT^).

### 2.3. Immunohistochemical staining (IHC) for the FN1 protein

The Ultra Sensitive SP IHC Kit (Maxim-Bio, Fuzhou City, Fujian Province, China) was used to detect the FN1 protein expression in ESCC tumor tissues and normal esophageal tissue according to the manufacturer protocol. The paraffin-embedded sections were deparaffinized, rehydrated. Antigen retrieval was accomplished by high pressure to the specimens in a 0.01 M citrate buffer for 8 minutes. Mouse anti-human fibronectin monoclonal antibody (Bio-Techne) optimum concentration was 15 μg/mL. PBS as a negative control instead of primary antibody. Two independent investigators evaluated tissue staining results in a blinded study. Classification of staining levels based on a semiquantitative IHC reference scale.^[13]^ The percentage of positive cells (P) is graded as: 0 for no stained cells; 1 score means ≤ 25% positive cells; 2 scores for 26% to 50% positive cells; 3 scores for ≥50% positive cells; and staining intensity (I): 1 score for light staining; 2 scores for moderate staining; and 3 scores for strong staining. The product of positive cell percentage grading and staining intensity ≥4 (P × I ≥ 4) means high expression, ≤4 means low expression, and a score of 0 means no expression.

### 2.4. Statistical analysis

SPSS Statistics for Windows (Version 19, SPSS) was used for statistical analysis. All experiments in the present study were repeated at least 3 times, and the data collected from 3 independent experiments are presented as the mean ± SD. The differences between the groups were assessed by Student *t* test. The categorical data were analyzed by a chi-square or Fisher exact test. Survival curves were plotted using the Kaplan–Meier method and were analyzed using the log-rank test. Univariate and multivariate survival analyses were performed using Cox proportional hazards regression model. All tests were 2-sided. *P* values <.05 were considered statistically significant.

## 3. Results

### 3.1. FN1 mRNA and protein expression levels in ESCC tissue samples

**Figure 1.**
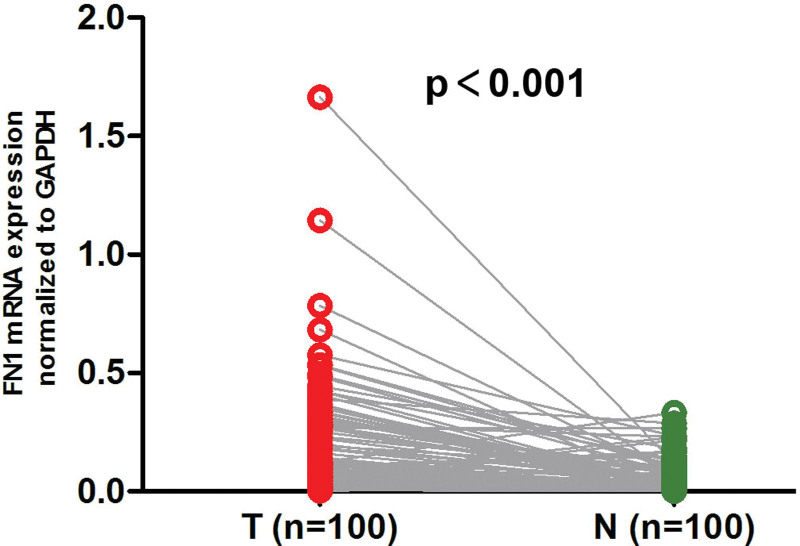
The expressions of FN1 mRNA in 100 pairs of ESCC tumor tissues and adjacent normal esophageal tissues were detected by qRT-PCR. FN1 mRNA expression in tumor tissues was significantly higher than that in the normal tissues. T, ESCC tumor tissue; N. adjacent normal esophageal tissue. *P* < .001. ESCC = esophageal squamous cell carcinoma, FN1 = fibronectin 1, qRT-PCR = quantitative real-time PCR.

The expression of FN1 mRNA in 100 pairs of ESCC tissues and adjacent normal esophageal tissues were detected by qRT-PCR. As shown in Figure 1, FN1 mRNA expression in ESCC tumor tissues was significantly higher than that in the corresponding normal esophageal tissues (*P* < .001).

**Figure 2.**
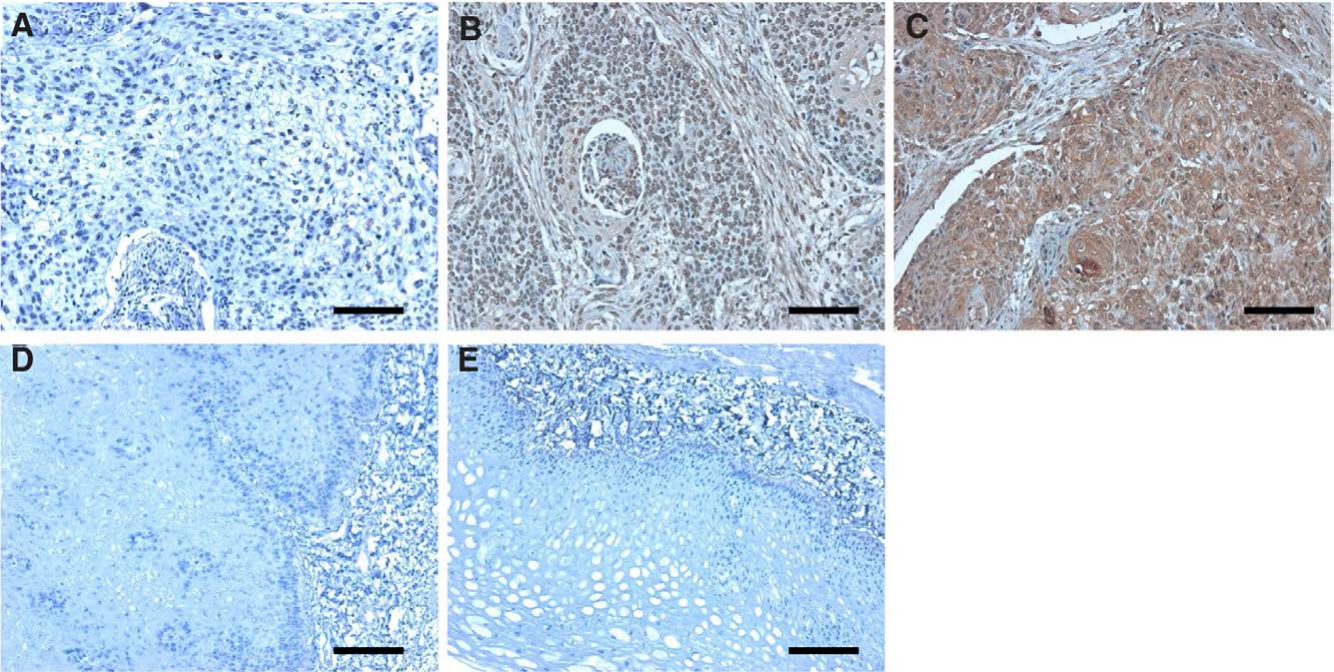
Immunohistochemical staining of FN1 protein in ESCC and normal esophageal epithelium tissues. (A) Negative FN1 protein expression in a representative ESCC tissue section. (B) Low FN1 protein expression in a representative ESCC tissue section. (C) High FN1 protein expression in a representative ESCC tissue section. (D) Negative FN1 protein expression in a representative epithelial tissue section of esophagus. (E) Weak FN1 expression in a representative epithelial tissue section of esophagus. Scale bars, 200 μm. ESCC = esophageal squamous cell carcinoma, FN1 = fibronectin 1.

FN1 protein expression was detected by IHC in 100 ESCC tumor specimens. FN1 protein was highly expressed in 66 (66.0%) of the tumor tissues. FN1 protein was expressed in tumor cells and stroma, localized in the membrane and cytoplasm of tumor cells (Fig. 2). In the epithelial basal layer, FN1 protein expression was low (Fig. 2). Rank correlation test was carried out for the expression degree of FN1 mRNA and the expression intensity of FN1 protein. The results showed that Pearson Correlation was 0.289, *P* = .003. The expression of FN1 mRNA and protein were positively correlated and the correlation was statistically significant.

### 3.2. Correlation analysis between patient characteristics and FN1 mRNA or FN1 protein expression

The correlation between FN1 mRNA expression in ESCC tissues and the clinicopathological characteristics of these 100 ESCC patients was analyzed. FN1 mRNA expression values above the median of all samples were considered as high expression, while samples below the median were low expression. Patients were classified by gender, age, smoking index, alcohol consumption index, degree of differentiation, tumor size, TNM stage and clinical stage. As shown in Table 1, increased expression of FN1 mRNA was positively correlated with smoking index, T classification, N classification and clinical stage (*P* = .028, *P* < .001, *P* = .001, and *P* < .001; respectively), but not with age, gender, drinking index, tumor location, tumor differentiation, tumor size and metastasis (*P* > .05).

**Table 1:** The association between FN1 mRNA expression and ESCC patient clinicopathological.

Characteristics features	FN1 mRNA		*P* value*
	Low expression (%)	High expression (%)	
Age (yr)			.069
<60	17 (17%)	27 (27%)	
≥60	33 (33%)	23 (23%)	
Gender			1.0
Male	48 (48%)	47 (47%)	
Female	2 (2%)	3 (3%)	
Smoking index			.028
Yes	40 (40%)	48 (48%)	
No	10 (10%)	2 (2%)	
Drinking index			.483
Yes	36 (36%)	40 (40%)	
No	14 (14%)	10 (10%)	
Tumor location			.771
Upper	2 (2%)	2 (2%)	
Middle	29 (29%)	27 (27%)	
Lower	19 (19%)	21 (21%)	
Differentiation			.454
Well + moderate	38 (38%)	42 (42%)	
Poor	12 (12%)	8 (8%)	
Tumor size (cm)			.156
≤3	25 (25%)	17 (17%)	
>3	25 (25%)	33 (33%)	
T classification			<.001
T1 + T2	33 (33%)	10 (10%)	
T3 + T4	17 (17%)	40 (40%)	
N classification			.001
N0	29 (29%)	12 (12%)	
N1 + N2 + N3	21 (21%)	38 (38%)	
M classification			1.0
M0	49 (49%)	49 (49%)	
M1	1 (1%)	1 (1%)	
Clinical stage			<.001
I + II	38 (38%)	15 (15%)	
III + IV	12 (12%)	35 (35%)	

ESCC = esophageal squamous cell carcinoma, FN1 = fibronectin 1.

* Chi-squared or Fisher exact tests.

According to the expression intensity of FN1 protein, patients were divided into 2 groups: low expression group (negative + low expression) and high expression group. As shown in Table 2, FN1 protein expression was positively correlated with tumor size, T classification, N classification, and clinical stage (*P* < .001, *P* < .001, *P* = .002, and *P* < .001, respectively). However, no significant correlation was found between FN1 protein and patient characteristics such as age, gender, smoking index, drinking index, tumor location, differentiation or M classification (*P* > .05).

**Table 2:** The association between FN1 protein expression and ESCC patient clinicopathological.

Characteristics features	FN1 protein		*P* value*
	Negative + low expression (%)	High expression (%)	
Age (yr)			.144
<60	13 (13%)	16 (16%)	
≥60	21 (21%)	50 (50%)	
Gender			.438
Male	31 (31%)	64 (64%)	
Female	3 (3%)	2 (2%)	
Smoking index			
Yes	26 (26%)	56 (56%)	.302
No	8 (8%)	10 (10%)	
Drinking index			.937
Yes	26 (26%)	50 (50%)	
No	8 (8%)	16 (16%)	
Tumor location			.168
Upper	1 (1%)	3 (3%)	
Middle	15 (15%)	41 (41%)	
Lower	18 (18%)	22 (22%)	
Differentiation			.795
Well + moderate	28 (28%)	52 (52%)	
Poor	6 (6%)	14 (14%)	
Tumor size (cm)			<.001
≤3	20 (20%)	13 (13%)	
>3	14 (14%)	53 (53%)	
T classification			<.001
T1 + T2	26 (26%)	17 (17%)	
T3 + T4	8 (8%)	49 (49%)	
N classification			.002
N0	21 (21%)	20 (20%)	
N1 + N2 + N3	13 (13%)	46 (46%)	
M classification			.786
M0	34 (34%)	64 (64%)	
M1	0 (0)	2 (2%)	
Clinical stage			<.001
I + II	30 (30%)	23 (23%)	
III + IV	4 (4%)	43 (43%)	

ESCC = esophageal squamous cell carcinoma, FN1 = fibronectin 1.

* Chi-square or Fisher exact tests.

### 3.3. FN1 mRNA or FN1 protein higher expression was correlated with poor survival, and FN1 protein expression was an independent prognostic factor

**Figure 3.**
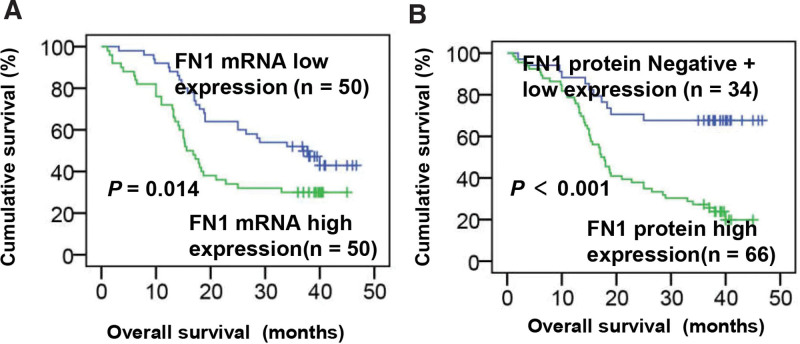
Kaplan–Meier survival analysis. (A) The correlation between FN1 mRNA expression and overall survival. Median expression level of FN1 mRNA was used as a cutoff to divide the 100 patients into low group (n = 50) and high group (n = 50). *P* = .014. (B) The correlation between FN1 protein expression and overall survival. Difference between the 2 groups were analyzed by log-rank test. *P* < .001. FN1 = fibronectin 1.

The 3-year overall survival rate of all 100 patients with ESCC was 35.0% and medium survival time was 23.64 months. Kaplan–Meier survival analysis displayed that ESCC patients with higher FN1 mRNA or FN1 protein expression had worse survival (*P* = .014 and *P* < .001; Fig. 3A and B). In the univariate analysis, higher FN1 mRNA and higher FN1 protein expression were found to be significant prognostic factors for poor survival (*P* = .016 and *P* < .001, respectively), in addition to T classification, N classification, and clinical stage (*P* = .017, *P* = .002, and *P* = .022, respectively; Table 3). Moreover, multivariate cox analysis of the 5 factors revealed that the N classification, clinical stage and FN1 protein higher expression were independent prognostic factors (*P* = .002, *P* = .030, and *P* = .003; Table 4).

**Table 3:** Univariate regression analysis of FN1 mRNA expression, FN1 protein expression and clinicopathological characteristics.

Clinicopathological characteristics	HR	95% CI	*P* value
Age (yr)(<60 vs ≥60)	1.040	0.629–1.719	.878
Gender (male vs female)	0.516	0.126–2.111	.357
Smoking index (yes vs no)	1.357	0.689–2.670	.377
Drinking index (yes vs no)	1.538	0.833–2.838	.169
Tumor location (upper, middle to lower)	1.205	0.776–1.872	.406
Differentiation (well + moderate vs poor)	0.899	0.488–1.659	.734
Tumor size (cm) (≤3 vs >3)	1.017	0.615–1.681	.948
T classification (T1 + T2 vs T3 + T4)	1.905	1.121–3.238	.017
N classification (N0 vs N1 + N2 + N3)	2.304	1.366–3.887	.002
M classification (M0 vs M1)	0.866	0.120–6.258	.887
Clinical stage (I + II vs III + IV)	1.801	1.090–2.977	.022
FN1 mRNA (Low vs high)	1.862	1.124–3.085	.016
FN1 protein (Negative + low vs high)	3.407	1.770–6.560	<.001

CI = confidence interval, FN1 = fibronectin 1, HR = hazard ratio.

**Table 4:** Multivariate cox regression analysis of FN1 expression and clinicopathological characteristics.

Characteristics features	HR	95% CI	*P* value
T classification (T1 + T2 vs T3 + T4)	1.469	0.714–3.02	.296
N classification (N0 vs N1 + N2 + N3)	3.502	1.584–7.743	.002
Clinical stage (I + II vs III + IV)	0.367	0.148–0.910	.030
FN1 mRNA (low vs high)	1.128	0.599–2.123	.710
FN1 protein (negative + low vs high)	3.129	1.484–6.597	.003

CI = confidence interval, FN1 = fibronectin 1, HR = hazard ratio.

### 3.4. The value of FN1 mRNA and FN1 protein as markers for the diagnosis of lymph node metastasis in ESCC

**Figure 4.**
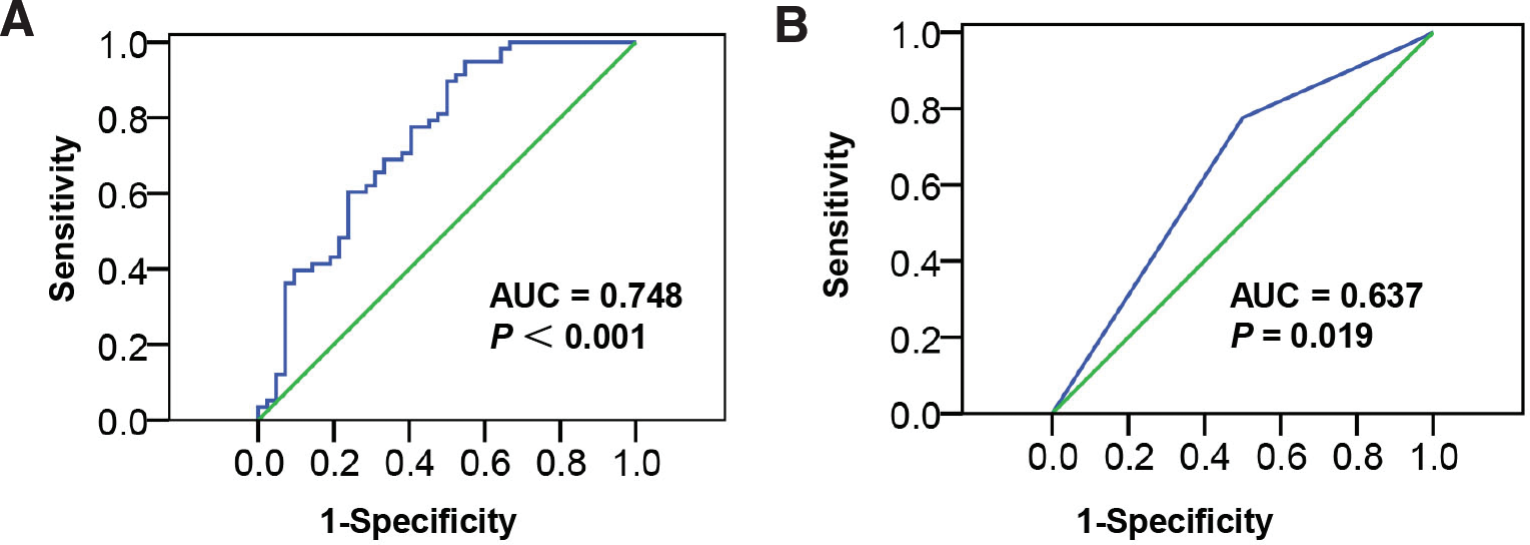
The ROC curves of FN1 mRNA and FN1 protein. (A) FN1 mRNA had better accuracy in distinguishing lymph node metastasis. *P* < .001. (B) FN1 protein had lower accuracy distinguishing lymph node metastasis. *P* = .019. FN1 = fibronectin 1.

ROC curve analysis was applied to assess whether FN1 mRNA or FN1 protein could be used as a marker for lymph node metastasis in ESCC. Figure 4 shows that the area under the curve values were 0.748 and 0.637. FN1 mRNA had better accuracy in determining lymph node metastasis (*P* < .001, Fig. 4A), while FN1 protein had lower accuracy (*P* = .019, Fig. 4B), suggesting that FN1 mRNA expression level may be a potential biomarker to distinguish whether ESCC has lymph node metastasis.

## 4. Discussion

EC is a highly lethal malignancy that ranks ninth in incidence and sixth in mortality among all malignancies in the world.^[1]^ In China, the main pathological type of EC is ESCC.^[3]^ FN1 is a large dimeric structural glycoprotein. FN1 has been reported to be overexpressed in certain tumor types, such as parathyroid cancer, hepatoblastoma,^[14]^ renal clear cell carcinoma,^[8]^ and ovarian cancer.^[15]^ However, the expression of FN1 protein in ESCC tumor tissues is still controversial.^[16]^

In the present study, we found that FN1 protein was expressed in both tumor cells and stroma. This result is different from Xiao et al,^[16]^ who found that FN1 protein was expressed in the tumor stroma of ESCC, but not in tumor cells. By analyzing the relationship between FN1 and clinicopathological factors, we found that the high expression of FN1 mRNA and FN1 protein in ESCC tumor tissues was significantly correlated with the depth of tumor invasion, lymph node metastasis and tumor clinical stage, and these clinical features were associated with tumor progression. FN1 mRNA was significantly correlated with smoking index and FN1 protein was significantly correlated with tumor size. patients with higher FN1 mRNA or FN1 protein expression had significantly lower survival rates than those with lower FN1 mRNA or FN1 protein expression. In addition, multivariate analysis showed that positive FN1 protein expression was an independent prognostic factor for poor survival. The ROC curve results showed that FN1 mRNA had better accuracy in differentiating lymph node metastases. This finding in human ESCC tumor specimens supports the notion that FN1 protein may play a role in ESCC progression and FN1 mRNA as a biomarker for determining lymph node metastasis.

Our study provides preliminary evidence that FN1 plays a pro-cancer role in ESCC, which is consistent with previous findings on the role of FN1 in different types of cancer. Wang et al^[17]^ reported that FN1 can promote invasion and migration of nasopharyngeal carcinoma cells. Recent reports have shown that FN1 expression is associated with lymph node metastasis in human cancers, including clear cell renal cell carcinoma,^[8]^ thyroid cancer^[18]^ and ESCC.^[19]^ FN1 and its receptors play an important role in mediating cell adhesion, migration and signaling and may limit the prevention of apoptosis in certain tissues.^[3]^ Increased activation of FN1 signaling has been associated with the promotion of epithelial-mesenchymal transition (EMT).^[20,21]^ The main characteristics of EMT are the destruction of intercellular contacts and the enhancement of cell motility, which enable cancer cells to invade surrounding tissues and then enter the circulation, thus promoting distant metastasis.^[22–24]^ FN1 may promote the process of EMT in human oral squamous cell carcinoma cells.^[21]^ Wang et al^[25]^ reported that the propagation of FN1 signaling pathway including FAK phosphorylation is critical to the promotion of EMT and EMT is associated with lymph node metastasis. Some studies have reported that FN1 stimulates the growth of human cancer cells by binding to the α5β1 integrin receptor on the cell surface and activating MEK1/ERK pathway^[26–29]^ suggests that activation of this pathway is associated with tumor development.

Our research initially demonstrated the value of measuring the intensity of FN1 mRNA or FN1 protein expression in postoperative tumor specimens from patients with ESCC in determining the prognosis of patients. However, further clinical studies are needed to determine its effectiveness in accurately determining the prognosis of patients. Whether FN1 can be used as a biomarker for lymph node metastasis requires more experiments to verify. At present, imaging evidence is still essential to determine whether lymph nodes are metastatic, while the expression intensity of FN1 mRNA or FN1 protein is only a secondary reference.

In summary, we have detected the expression of FN1 in ESCC patients by qRT-PCR and IHC. This study found that FN1 protein is expressed in tumor cells and stroma. High expression of FN1 protein is an independent prognostic factor of poor survival, and it could become a potential target for clinical treatment of ESCC.

## Author contributions

**Investigation:** Min Su.

Methodology: Min Su.

Resources: Shaolin Chen.

Software: Wenxiang Wang.

Supervision: Wenxiang Wang.

Writing – original draft: Junliang Ma.

Writing – review & editing: Shaolin Chen.
